# Tobacco-Related Mortality among Persons with Mental Health and Substance Abuse Problems

**DOI:** 10.1371/journal.pone.0120581

**Published:** 2015-03-25

**Authors:** Frank C. Bandiera, Berhanu Anteneh, Thao Le, Kevin Delucchi, Joseph Guydish

**Affiliations:** 1 University of Texas School of Public Health, Dallas, Texas, United States of America; 2 Addictions and Mental Health Division, Oregon Health Authority, Salem, Oregon, United States of America; 3 Institute for Health Policy Studies, University of California San Francisco, San Francisco, California, United States of America; Peking University, CHINA

## Abstract

The rate of cigarette smoking is greater among persons with mental health and/or substance abuse problems. There are few population-based datasets with which to study tobacco mortality in these vulnerable groups. The Oregon Health Authority identified persons who received publicly-funded mental health or substance abuse services from January 1996 through December 2005. These cases were then matched to Oregon Vital Statistics records for all deaths (N= 148,761) in the period 1999-2005. The rate of tobacco-related death rates was higher among persons with substance abuse problems only (53.6%) and those with both substance abuse and mental health problems (46.8%), as compared to the general population (30.7%). The rate of tobacco-related deaths among persons with mental health problems (30%) was similar to that in the general population. Persons receiving substance abuse treatment alone, or receiving both substance abuse and mental health treatment, were more likely to die and more likely to die prematurely of tobacco-related causes as compared to the general population. Persons receiving mental health services alone were not more likely to die of tobacco-related causes, but tobacco-related deaths occurred earlier in this population.

## Introduction

Tobacco remains the leading behavioral risk factor for chronic disease morbidity and mortality. There is a well-established association between cigarette smoking and mental disorders (e.g., major depressive disorder and schizophrenia),[1] with 36% of persons with mental health diagnoses versus just 19% of the United States (U.S.) general population being smokers.[2] Similarly, persons who abuse alcohol, marijuana or hard drugs are likely to smoke cigarettes,[3–6] with 70% of persons who received past year treatment for substance abuse being smokers.[7] Persons with mental health and substance abuse problems start smoking at a younger age[8] and have higher levels of dependence,[9, 10] and persons with mental illness smoke 45% of cigarettes in the U.S.[9] Smokers who are mentally ill have higher rates of psychiatric symptoms, more psychiatric hospitalizations and poorer treatment outcomes.[11–13] There are also differences in health behaviors, such as smoking, by gender and socioeconomic status. Traditionally, men have smoked at higher rates than women, although this gap has narrowed[14]. Nevertheless, men are more likely to die from tobacco-related causes than women[14]. There are also differences in unhealthy behaviors by socioeconomic status, with persons of a lower socioeconomic status having higher rates of smoking than persons of higher socioeconomic status[14]. Lower socioeconomic status may also affect access to health insurance, including access to mental health and substance abuse services. For example, over two thirds of all drug abuse treatment is provided in the public sector[15]. Thus, considering gender and socioeconomic status when studying tobacco-related deaths among persons with mental health and substance abuse problems is important.

Although persons with mental illness have higher rates of suicide, they also have high rates of all-cause morbidity and mortality. McCarrick et al.[16] studied 1,471 persons from the community and found that persons with mental illness had high rates of chronic diseases, such as heart disease, respiratory disease, cancers, and diabetes. Persons with depression are likely to die from heart disease,[17] respiratory disease,[18] and cancer. McGinty et al.[19] sampled 3,317 Maryland Medicaid adult beneficiaries with schizophrenia or bipolar disorder from 1994 through 2004. Using ICD-9 codes and aggregating all cancers (i.e., breast, lung, colorectal and prostate), they found that cancer incidence for adults with schizophrenia or bipolar disorder was 2.6 higher than the general population, a finding they attributed partly to smoking.

Attention has also been paid to premature mortality among psychiatric patients.[20–26] Colton and Manderscheid [27] conducted a study in 8 states in the U.S. and found that persons with mental illness died on average 25 years before the general population. Although this estimate of premature death by 25 years is widely cited,[1, 28–32] other studies have found smaller estimates of years of life lost, more than or equal to 8 years.[20–26] While they die younger than the general population, persons with mental illness die from the same causes of death as the general population. [27, 33] Lawrence et al.[33] studied premature mortality in persons with mental health or substance abuse problems in Western Australia from 1985 to 2005. They found that almost 80% of deaths among these persons are attributed to chronic diseases.[33] It has been postulated that one reason for the health disparity between persons with mental illness and the general population includes preventable lifestyle factors such as smoking, leisure time activity, poor diet and alcohol abuse in addition to not seeking medical care, psychiatric medication use, high risk sex practices, and poverty.[27, 33]

Persons with substance abuse problems are likely to die prematurely from suicide, homicide, and drug overdoses.[34] They are also likely to die from chronic diseases, including cancers.[22] At least two studies suggest that tobacco may contribute to premature death among persons with substance abuse problems. Hser et al. identified 405 patients admitted to the California Civil Addict Program from 1962 through 1964, and followed them to 1986. Smokers in this sample were four times more likely to die than were non-smokers.[35] Similarly, Hurt et al. identified 845 participants who received inpatient treatment for alcohol dependence in Olmsted County, Minnesota between 1972 and 1983, and located death records for this sample up to 1994. Persons in this sample were more likely to die from tobacco-related causes than from alcohol-related causes.[36]

None of these studies of the role of tobacco use in the deaths of persons with mental health and/or substance abuse problems used large population-based datasets where it is possible to include a general population comparison group. The current study used unique statewide data from a previous state report in Oregon,[37] in which persons who were treated for mental health and/or substance abuse between 1996 and 2005 were matched with death records from 1999 to 2005. Oregon death records include physician assessment of whether the death was tobacco-related, a validated measure[38–40] of tobacco-related death. The National Center for Health Statistics has recommended that this measure be added to death certificates in all states; however, only a few states have complied. Physician assessment at time of death offers a direct and interpretable measure of whether deaths are tobacco-related. We hypothesized that persons with mental health and/or substance abuse problems would have elevated rates of tobacco-related deaths, compared to the general population. We also hypothesized that these persons would die younger from tobacco-related deaths.

## Materials and Methods

### Study Design

Using Oregon's statewide Client Process and Monitoring System (CPMS), the Oregon Health Authority Addictions and Mental Health (AMH) Division identified Oregonians who received publicly funded treatment for mental health and/or substance abuse problems from January 1996 through December 2005. Patients did not consent to have their information used for study purposed but the data were de-identified. The CPMS collects demographic characteristics and diagnostic information on each treatment episode. CPMS records were matched by the Oregon Health Authority AMH Division with death records from vital statistics, representing all deaths recorded in Oregon for 1999–2005. We analyzed death records in four groups: 1) General population (n = 136,827), 2) persons with mental health problems only (MHO, n = 8,789), 3) persons with substance abuse problems only (SAO, n = 2,244) and 4) persons with both mental health and substance abuse problems (dual, n = 901). Study procedures used only de-identified data, involved no contact with human participants, and were certified as exempt from review by the University of California, San Francisco Institutional Review Board.

### Measures

#### Demographics

Age at death, gender, education (< high school, > = high school, and some college), and race (White, Black, or other) were obtained from the death certificates.

#### Tobacco-related deaths

Tobacco-related deaths are often measured using mathematical models using ICD-9 or ICD-10 codes, reflecting cancers, cardiovascular disease, and other conditions or causes associated with tobacco use. Use of ICD-9 and ICD-10 codes have been criticized, however, as leading to under-estimates of tobacco-related deaths^31–33^ and the CDC has recommended that physicians use specific mention of whether the death was tobacco-related in death certificates. This places the determination of tobacco-related death with the physician completing the death certificate, and enabling the physician to use any data available at the time of death concerning tobacco as a contributing factor. Previous studies have shown that physician report is a valid measure of tobacco-related mortality.[38–40]

Since January 1989, Oregon has included the question “Did tobacco contribute to this death?” Possible responses were Yes, Probably, No, and Unknown. An “unknown” response does not indicate missing data but, rather, that the certifier actively reported that the role of tobacco in the certified death was unknown or undetermined when the form was completed. As in previous validated studies,[38–40] responses “Yes” or “Probably” were considered as positive and “No” as negative. The health division in Oregon routinely contacts the certifying physician when a box is not checked or the response does not seem logical. We defined this question as physician reported tobacco attributable deaths.

### Statistical analyses

The complete dataset included all deaths recorded in Oregon from 1999 through 2005 (N = 208,622). As we were interested in natural deaths where tobacco may be a contributing factor, we removed 17,398 records where the cause of death was accident, suicide, homicide, pending, undetermined, or missing. We also removed 1,022 records where question about the role of tobacco was not completed. The remaining dataset included 190,202 records for natural deaths where the physician assessment of tobacco-related death was completed. These records included 148,761 records (78%) where the role of tobacco in the death was known (physicians reported the death as definitely, probably, or not at all tobacco-related), and 41,441 (22%) where the role of tobacco was unknown.

Cases where the tobacco-related nature of the death was coded by the certifying physician as “unknown” offer challenge to analysis and interpretation. This is not missing data, because the physician applied a code suggestive of uncertainty or insufficient information. To assess potential bias that the unknown cases may have on results, we first describe available demographic characteristics using means and percentages, and compare these characteristics for cases where the tobacco-related death status was known to those where it was unknown. As these are population data where small differences may achieve statistical significance, we evaluate the difference between groups using effect size. For descriptive purposes only, we present the distribution of deaths by ICD-10 codes for the four largest causes of death (i.e., neoplasms, cardiovascular disease, respiratory disease, diabetes), both for the full sample and for the cases where tobacco-related death status was known.

Comparison of demographic characteristics for cases where the role of tobacco in the death was known vs. unknown were very small. Following the convention applied in previous studies using Oregon death records to study tobacco use,[38–40] the remaining analyses focused on the 148,761 records where the role of tobacco was known. For these cases, we report deaths where the role of tobacco was recorded on the death certificate as yes (combining “yes” and “probably”) or no, and for each of the four study groups (general population, MHO, SAO, dual). To assess whether persons with MHO or SAO died prematurely from tobacco, we stratified tobacco-related deaths (n = 46, 209) for each group into five age categories: < 50 years, 50–59 years, 60–69 years, 70–79 years and ⩾ 80 years. To assess whether the pattern of tobacco-related mortality may differ by gender, we present the same breakdown stratified by gender. To assess how inclusion of the unknown cases may affect findings, we also report proportions of tobacco-related deaths in each category while including unknown cases in the denominator. The dataset includes all deaths in Oregon for the period under study. It is not a sample, and no inferences are drawn concerning other populations, so the use of inferential statistics is unnecessary.

Last, we used multiple logistic regression to calculate odds ratios (ORs) to describe tobacco-related deaths in each group, and while controlling for age at death, gender, race and education. We also explore the role of race, education, and gender as covariates and report their odds of tobacco-related deaths. The physician assessment of whether or not a death is tobacco-related is a direct attribution made by a medical professional at the time of death, so no attributable death calculations were performed.

## Results

### Demographic characteristics

Demographic characteristics of the population, and comparison for groups where tobacco-related deaths were known or unknown, are shown in Table 1. Mean age of death where the role of tobacco was known was 77.2 (sd = 14.0), compared to 77.0 (sd = 14.0) where the role of tobacco was unknown. As mean values may obscure some differences, Table 1 reports the proportions of person with tobacco-related deaths known or unknown in 5 age categories. For all cases combined (combined data not shown in table), 51.7% of deaths occurred over age 80, 96.8% were White, 52.7% were women. Also for all cases combined, most (91.5%) were classified in the general population, while smaller proportions were classified as MHO (6.2%), SAO (1.6%), or dual problem (0.07%; combined data not shown in table). The third column shows effect sizes for the comparison between the cases where tobacco-related deaths where coded as known to those where tobacco-related deaths were coded as unknown. All effect sizes shown in the table are below 0.10, which Cohen[41] identified as a small effect. This suggests that, for age, race, gender, and classification into study group, cases where the role of tobacco was unknown were very similar to cases where the role of tobacco was known. Remaining analyses focus on the 148,761 cases where the role of tobacco in the death was coded on the death certificate as yes (combining definitely or probably) or no.

**Table 1:** Demographic characteristics for cases with natural deaths occurring in Oregon State, 1999–2005.

	Natural deaths (N = 190,202)*			Tobacco related death ** was known (N = 148,761)		
	Tobacco related death was known (n = 148,761)	Tobacco related death was unknown (N = 41,441)	Effect size	Related to tobacco (n = 46,209)	Not related to tobacco (n = 102,552)	Effect size
Age at death			0.018			0.264
<50	7,034 (4.7%)	2,183 (5.3%)		1,615 (3.5%)	5,419 (5.3%)	
50–59	11,337 (7.6%)	3,304 (8.0%)		4,687 (10.1%)	6,650 (6.5%)	
60–69	18,372 (12.4%)	4,850 (11.7%)		9,141 (19.8%)	9,231 (9.0%)	
70–79	35,430 (23.8%)	9,350 (22.6%)		15,610 (33.8%)	19,820 (19.3%)	
≥80	76,588 (51.5%)	21,754 (52.5%)		15,156 (32.8%)	61,432 (59.9%)	
Race			0.017			0.019
White	144,241 (97.0%)	39,964 (96.4%)		44,967 (97.3%)	99,274 (96.8%)	
Black	1,653 (1.1%)	643 (1.6%)		531 (1.2%)	1,122 (1.1%)	
Other	2,867 (1.9%)	834 (2.0%)		711 (1.5%)	2,156 (2.1%)	
Gender			0.054			0.164
Male	68,295 (45.9%)	21,759 (52.5%)		26,934 (58.3%)	41,361 (40.3%)	
Female	80,466 (54.1%)	19,682 (47.5%)		19,275 (41.7%)	61,191 (59.7%)	
Education			0.020			0.082
< High school	34,879 (23.7%)	10272 (25.2%)		12,012 (26.4%)	22,867 (22.6%)	
= High school	62,701 (42.7%)	17,624 (43.3%)		20,845 (45.8%)	41,856 (41.3%)	
> High school	49,036 (33.4%)	12,736 (31.3%)		12,573 (27.6%)	36,463 (36.0%)	
Population			0.035			0.066
Mental health only	8,789 (5.9%)	3,051 (7.4%)		2,635 (5.7%)	6,154 (6.0%)	
Substance abuse only	2,244 (1.5%)	820 (2.0%)		1,203 (2.6%)	1,041 (1.0%)	
Dual problems	901 (0.6%)	394 (1.0%)		422 (0.9%)	479 (0.5%)	
General population	136,827 (92.0%)	37,176 (89.7%)		41,949 (90.8%)	94,878 (92.5%)	
Tobacco-related deaths						
Yes	46,209 (31.1%)					
No	102,552 (68.9%)					

* Excludes 17,398 records where the cause of death was unnatural (e.g., accident, suicide, homicide), and 1,022 cases where the role of tobacco use in the death was coded as missing.

** Includes 46,209 cases where the role of tobacco in the death was coded as “definitely” or “probably,” and 102,552 where the role of tobacco was coded as “no.”

Also shown in Table 1 are the distributions of age, race, and gender between deaths related to tobacco and those not related to tobacco. As observed in the general literature persons who died from tobacco-related causes were more likely to die younger than persons who did not die from tobacco-related causes. For example, 19.8% of tobacco-related deaths occurred in the 60–69 age group, while only 9% of all other deaths occurred in this same age group. There were only small differences in race between tobacco-related and non-tobacco-related deaths (effect size = .019). Last, as observed in the general literature males were more likely to die from tobacco deaths than females (effect size = .164).

Table 2 summarizes the most common ICD-10 natural causes of deaths. In all natural deaths (N = 190,202), approximately 37% of deaths were from the circulatory system, 26% were from neoplasms, 10% were from the respiratory system, and 3% were from diabetes. In natural deaths were tobacco deaths were known (N = 148,761), approximately 35% of deaths were from the circulatory system, 28% were from neoplasms, 11% were from the respiratory system, and 3% were from diabetes.

**Table 2:** Distribution of most common ICD-10 deaths for cases with natural deaths occurring in Oregon State, 1999–2005.

All natural deaths* (N = 190,202)		Tobacco related death ** was known(N = 148,761)	
	**%**		**%**
Neoplasm	26.0	Neoplasm	28.1
Circulatory System	37.0	Circulatory System	34.8
Respiratory System	10.2	Respiratory System	11.1
Diabetes	3.5	Diabetes	3.4
Others	23.3	Others	22.6

* Excludes 17,398 records where the cause of death was unnatural (e.g., accident, suicide, homicide), and 1,022 cases where the role of tobacco use in the death was coded as missing.

** Includes 46,209 cases where the role of tobacco in the death was coded as “definitely” or “probably,” and 102,552 where the role of tobacco was coded as “no.”

### Mortality among persons with mental health and/or substance abuse problems associated with tobacco use

**Fig 1:**
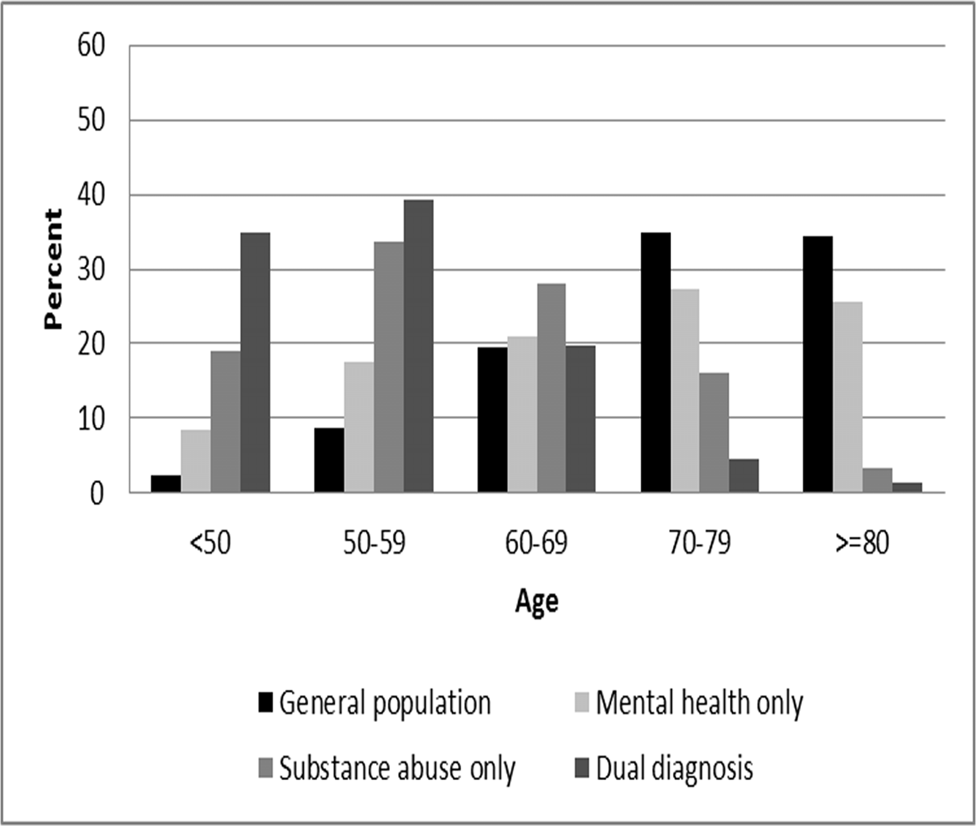
Tobacco-related deaths by age for Oregonians with mental health and/or substance abuse problems, 1999–2005 (N = 46,209).

The proportion of tobacco-related deaths in each group (general population, MHO, SAO, dual) is reported in Table 3. The first column shows that 30.7% of deaths in the general population were coded as tobacco-related, compared to 30% in the MHO group, 53.6% in the SAO group, and 46.8% in the dual services group. The remaining columns in Table 3 show the distribution of tobacco-related deaths across age groups. For example, in the general population 2.4% of tobacco-related deaths occurred under age 50 and 34.4% occurred at age 80 or above. Transferring these proportions to a bar graph offers a visual interpretation (see Fig. 1). Under age 60, and compared to the general population, tobacco-related deaths were successively higher in the MHO group, the SAO group, and the dual services group. That is, persons who received mental health, substance abuse, or both types of services experienced tobacco-related deaths prematurely, as compared with those in the general population. At ages 60–69, tobacco-related deaths are similar in the general population, MHO and dual group, although still elevated in the SAO group (28%). After age 70 the pattern is reversed, such that tobacco-related deaths occur more frequently in the general population than in any other group.

**Table 3:** Tobacco-related deaths for persons receiving mental health and/or substance abuse services in Oregon State, 1999–2005 (N = 148,761) *.

	All deaths (N = 148,761)	Tobacco-related deaths by age group (N = 46,209)				
		<50	50–59	60–69	70–79	> = 80
	%	%	%	%	%	%
General population (n = 136,827)	30.7	2.4	8.7	19.5	35.0	34.4
Mental health only (n = 8,789)	30.0	8.5	17.5	21.0	27.4	25.6
Substance abuse only (n = 2,244)	53.6	19.1	33.6	28.0	16.0	3.3
Dual services (n = 901)	46.8	34.8	39.3	19.7	4.7	1.4

* Includes 46,209 cases where the role of tobacco in the death was coded as “definitely” or “probably,” and 102,552 where the role of tobacco was coded as “no.”

We recalculated what the proportion of tobacco-related deaths would be for each study group under the most conservative condition. For each group, we took the number of tobacco-related deaths and divided by the number of all other deaths (those where deaths were coded as not related to tobacco plus those where the role of tobacco was unknown). This offers an estimate of the proportion of deaths that are tobacco-related in each group, assuming that all deaths where the role of tobacco was unknown were not tobacco-related. For the general population, MHO, SAO and dual groups as reported in Table 1, and using only cases where the tobacco status was known (n = 148,761), the proportion of deaths that are tobacco-related are, respectively, 30.7%, 30%, 53.6% and 46.8%. When recalculated under the most conservative assumption (n = 190,202) these proportions were 24.1%, 22.2%, 39.2% and 32.5%. While the proportions are smaller, the relationship between them remains the same.

The proportion of tobacco-related deaths by gender in each group (general population, MHO, SAO, dual) is reported in Table 4. The first column shows that 39.0% of deaths among males in the general population were tobacco-related, whereas 23.6% of deaths among women in the general population were tobacco-related. These proportions are similar for the MHO group. Tobacco-related deaths, as a proportion of all deaths for men or for women, are closer in the SAO group (53.9% and 52.3%) and in the Dual group (48.1% and 44.7%). While tobacco is associated with a greater proportion of deaths among men than women in the general population and in the MAO group, tobacco is associated with very similar proportions of deaths for both men and women in the SAO and dual groups. Notably, tobacco-related deaths are higher in women than in men for those in the SAO group under 50 years of age. And tobacco-related deaths are higher in women than in men for those in the dual group under age 60.

**Table 4:** Tobacco-related deaths by gender for persons receiving mental health and/or substance abuse services in Oregon State, 1999–2005 (N = 148,761) *.

		All deaths (N = 148,761)	Tobacco-related deaths by age group (N = 46,209)				
			<50	50–59	60–69	70–79	> = 80
		%	%	%	%	%	%
General population	Male	39.0	2.8	7.5	19.8	34.5	33.1
	Female	23.6	1.8	7.4	18.9	35.5	36.1
Mental health only	Male	37.7	9.2	17.6	20.1	27.4	25.4
	Female	24.8	7.8	17.3	21.7	27.4	25.6
Substance abuse only	Male	53.9	15.7	34.6	29.3	16.7	3.5
	Female	52.3	32.9	29.0	22.6	12.8	2.5
Dual services	Male	48.1	33.0	36.4	23.0	5.9	1.4
	Female	44.7	37.9	44.4	13.7	2.6	1.3

* Includes 46,209 cases where the role of tobacco in the death was coded as “definitely” or “probably,” and 102,552 where the role of tobacco was coded as “no.”

The odds of tobacco-related death in each group (Table 5), relative to that in the general population and controlling for age at death, gender, race and education were 0.95 [95% confidence interval (CI): 0.90–1.00] for MHO, 1.82 [95% CI: 1.66–2.00] for SAO, and 1.88 [95% CI: 1.63–2.18] for those receiving both types of services. Men were more likely to die from tobacco-related deaths than women, 1.86 [95% CI: 1.82–1.90]; and persons of other race, 0.58 [95% CI: 0.53–0.63] and Blacks. 0.82 [95% CI: 0.73–0.92] were less likely to die from tobacco-related deaths than Whites; persons with < high school education, 1.77 [95% CI: 1.72–1.83] and high school education, 1.59 [95% CI: 1.55–1.63] were more likely to die from tobacco-related deaths than persons > high school education.

**Table 5:** Odds ratio estimates for demographics and vulnerably populations for tobacco-related deaths. (N = 148,761) *.

	Odds ratio	95% Confidence interval
Age		
> = 80	1.00	0.94–1.06
70–79	**3.06**	**2.8–3.2**
60–69	**3.83**	**3.59–4.10**
50–59	**2.69**	**2.51–2.88**
<50	Reference	
Gender		
Male	**1.86**	**1.82–1.90**
Female	Reference	
Race		
Other	**0.58**	**0.53–0.63**
Black	**0.82**	**0.73–0.92**
White	Reference	
Education		
< High school	**1.77**	**1.72–1.83**
= High school	**1.59**	**1.55–1.63**
> High school	Reference	
Population		
Mental health only	**0.95**	**0.90–1.00**
Substance abuse only	**1.82**	**1.66–2.00**
Dual services	**1.88**	**1.63–2.18**
General population	Reference	

* Includes 46,209 cases where the role of tobacco in the death was coded as “definitely” or “probably,” and 102,552 where the role of tobacco was coded as “no.”

## Discussion

While large epidemiologic studies have examined mortality among persons with mental illness^18–25^ and smaller studies have examined tobacco-related deaths among persons with substance abuse problems,^33–34^ this is the first study of tobacco-related deaths among persons with mental health or substance abuse problems using population based data and including a general population comparison. First, as compared to the general population, tobacco-related deaths are much higher among persons who recently received substance abuse services alone or in combination with mental health services. Second, persons recently receiving mental health services were not more likely to die of tobacco-related causes than were those in the Oregon general population. There were some gender differences observed in tobacco-related deaths. In the general population and mental health only population, tobacco-related deaths were higher among men than women; however, in both substance abuse only and dual services tobacco-related deaths were high in both men and women. Third, while tobacco-related premature deaths were most prominent among persons receiving both substance abuse and mental health services, all three groups (MHA, SAO, dual) experienced tobacco-related deaths at earlier ages than those in the general population. In addition, when exploring the role of gender, education, and race as covariates, we found that males, those of a lower education and Whites had greater odds for tobacco-related deaths, as observed in other studies[14].

While public health and tobacco control efforts have contributed to significant reductions in smoking at the population level, smoking prevalence remains high in substance abuse and mental health populations. Since Dreher and Fraser reported elevated rates of smoking among persons in alcohol treatment in 1967,[42] literature reviews[7] and analyses of state treatment data[43] and national epidemiologic data[7] demonstrate very high smoking prevalence in substance abuse treatment populations. Much less has been reported, however, about how elevated smoking prevalence is related to downstream morbidity and mortality in this population.[35]^,^ [36] Oregon data reported in the present study offer a direct estimate that over half of all natural deaths among those who received substance abuse treatment are tobacco-related deaths.

More has been written about the disproportionate burden of illness associated with tobacco use in mental health populations. [10, 32] Literature in this area often makes the commonsense assumption that, since persons with mental illness both smoke at a higher rate and die sooner than the general population, smoking causes at least some excess mortality.[28–32]

Oregon data do not support this conflation, since persons receiving mental health services did not die of tobacco-related deaths in greater proportion than the general population. Further research may confirm or disconfirm this finding but, until that time, caution is needed to interpret how higher smoking prevalence may be related to greater mortality among those with mental illness.

Last, Fig. 1 shows a pattern of premature tobacco-related death for persons with mental health, substance abuse, or both types of problems in relation to the general population. Persons in each of these groups who die of tobacco-related causes more often do so before age 60, while persons without these problems who die of tobacco-related causes more often do so after age 70. While persons with substance abuse problems suffer both excess and premature tobacco-related mortality, Oregon data suggests that those with mental health problems suffer premature, but not excess, tobacco-related mortality. In the general population and in the mental health population, the proportion of deaths that were tobacco-related was lower among women than among men. However, in the substance abuse and dual populations these proportions were very similar by gender and, for deaths occurring under age 50, were higher among women than among men.

Study limitations reflect challenges of using administrative datasets to explore questions for which the data were not intended. In this study, Oregon State agencies matched service records from 1996–2005 with death records from 1999–2005, and alternative approaches may have been to match records within the same time period, or to follow individual cases for the same length of time. We also used data based on records matched up to 2005, so that findings reported here are based on data nearly 10 years old. Nevertheless, there are few datasets available which permit direct observation of tobacco-related deaths for persons known to have substance abuse or mental health problems, and using population data.

In addition, 22% of cases were coded by the certifying physician as unknown, reflecting uncertainty about the possible role of tobacco in the death. Comparison of cases where the role of tobacco was known or unknown, using available demographic characteristics, showed only small differences. Similarly, comparing the proportion of deaths where the role of tobacco was known or unknown within each study group also showed small differences. That the distributions of these variables were similar in the known and unknown cases supports the assumption that tobacco-related deaths were also similar in the known and unknown cases. If so, then the estimates that the rate of tobacco-related deaths in the general population and MHO, SAO and dual groups were 30.7%, 30%, 53.6% and 46.8%, respectively, is sound. However, even the most conservative assumption that no unknown cases were tobacco-related resulted in lower proportions in the same pattern across the 4 groups, as above, 24.1%, 22.2%, 39.2% and 32.5%. This relationship was also observed when calculating odds of tobacco-related death in each group while controlling for other characteristics. We conclude that, for the four groups studied, the burden of tobacco-related mortality is greatest among persons who received substance abuse services, and among those who received both substance abuse and mental health services.

The dataset we studied did not include what substances (e.g., marijuana, alcohol, cocaine), or what types of mental disorders (e.g., schizophrenia and bipolar) persons were treated for. Our sample only included persons who received public treatment for mental health and/or substance abuse problems and thus did not include treatment from the private sector. It is then possible that these persons may have had more severe mental disorders and substance dependence. Thus, results may not generalize to the complete mental health and substance abuse populations in Oregon. Our sample was mostly comprised of White persons, consistent with the population of Oregon, limiting generalizability to ethnic or racial minority populations. The observation period in this study was short, matching cases receiving mental health or substance abuse services during 1996–2005 to all Oregon death records for the period 1999–2005, and a longer observation period may achieve different findings. Last, our analysis excluded suicides, homicides, and accidental deaths which are major contributors to premature mortality among persons with MHO, SAO and dual problems. However Pirie et al.[44] has shown that smoking is a leading cause of death, beyond these variables, among women in the United Kingdom (UK).

## Conclusions

This study provides findings of tobacco-related mortality among persons with mental health and/or substance abuse problems using large public health datasets. Findings show nearly half of all deaths were tobacco-related for persons who received substance abuse services, or who received both substance abuse and mental health services. Tobacco-related deaths in these populations, and in those who received mental health services only, occurred earlier by decades than that in the general population. These findings should drive addiction and mental health treatment programs and systems, state and federal agencies supporting such treatment, and tobacco control and regulatory authorities to address tobacco use in these populations. Tobacco use and dependence should be treated aggressively in behavioral health settings, and such treatment should include both counseling and medication interventions.[45] Beginning in 2014, Medicaid mandates coverage of tobacco cessation prescription drugs,[46] and this may increase access to cessation supports in behavioral health settings. Provider incentives have been associated with increased delivery of tobacco cessation services in the UK,[47] and may be considered in the U.S. particularly for clinical populations where smoking is prevalent. Behavioral health treatment programs and treatment systems should implement tobacco free policies designed to reduce, denormalize, and treat tobacco dependence, as has been done in hospital settings,[48] in VA Medical Centers,[49] and in the New York State addiction treatment system.[50] Additional and innovative tobacco control strategies are needed to address prevalent smoking, tobacco-related health disparities, and tobacco-related premature and excess mortality in behavioral health populations.

## Supporting Information

S1 Dataset Tobacco-related mortality dataset among substance abuse and mental health populations. (XLSX)
